# Persistent symptoms and decreased health-related quality of life after symptomatic pediatric COVID-19: A prospective study in a Latin American tertiary hospital

**DOI:** 10.6061/clinics/2021/e3511

**Published:** 2021-11-17

**Authors:** Thais T. Fink, Heloisa H.S. Marques, Bruno Gualano, Livia Lindoso, Vera Bain, Camilla Astley, Fernanda Martins, Denise Matheus, Olivia M. Matsuo, Priscila Suguita, Vitor Trindade, Camila S.Y. Paula, Sylvia C.L. Farhat, Patricia Palmeira, Gabriela N. Leal, Lisa Suzuki, Vicente Odone, Magda Carneiro-Sampaio, Alberto José S. Duarte, Leila Antonangelo, Linamara R. Batisttella, Guilherme V. Polanczyk, Rosa Maria R. Pereira, Carlos Roberto R. Carvalho, Carlos A. Buchpiguel, Ana Claudia L. Xavier, Marilia Seelaender, Clovis Artur Silva, Maria Fernanda B. Pereira, HC-FMUSP Pediatric Post-COVID-19 Study Group, Adriana M. E. Sallum, Alexandra V. M. Brentani, Álvaro José S. Neto, Amanda Ihara, Andrea R. Santos, Ana Pinheiro M. Canton, Andreia Watanabe, Angélica C. dos Santos, Antonio C. Pastorino, Bernadette D. G. M. Franco, Bruna Caruzo, Carina Ceneviva, Carolina C. M. F. Martins, Danilo Prado, Deipara M. Abellan, Fabiana B. Benatti, Fabiana Smaria, Fernanda T. Gonçalves, Fernando D. Penteado, Gabriela S. F. de Castro, Guilherme S. Gonçalves, Hamilton Roschel, Ilana R. Disi, Isabela G. Marques, Inar A. Castro, Izabel M. Buscatti, Jaline Z. Faiad, Jarlei Fiamoncini, Joaquim C. Rodrigues, Jorge D. A. Carneiro, Jose A. Paz, Juliana C. Ferreira, Juliana C. O. Ferreira, Katia R. Silva, Karina L. M. Bastos, Katia Kozu, Lilian M. Cristofani, Lucas V. B. Souza, Lucia M. A. Campos, Luiz Vicente R. F. Silva, Marcelo T. Sapienza, Marcos S. Lima, Marlene P. Garanito, Márcia F. A. Santos, Mayra B. Dorna, Nadia E. Aikawa, Nadia Litvinov, Neusa K. Sakita, Paula V. V. Gaiolla, Paula Pasqualucci, Ricardo K. Toma, Simone Correa-Silva, Sofia M. Sieczkowska, Marta Imamura, Silvana Forsait, Vera A. Santos, Yingying Zheng

**Affiliations:** Hospital das Clinicas HCFMUSP, Faculdade de Medicina, Universidade de Sao Paulo, Sao Paulo, SP, BR.; Hospital das Clinicas HCFMUSP, Faculdade de Medicina, Universidade de Sao Paulo, SP, BR.

**Keywords:** Long Coronavirus Disease 2019, Child, Adolescent, Sequelae, Multisystem Inflammatory Syndrome in Children

## Abstract

**OBJECTIVES:**

To prospectively evaluate demographic, anthropometric and health-related quality of life (HRQoL) in pediatric patients with laboratory-confirmed coronavirus disease 2019 (COVID-19)

**METHODS:**

This was a longitudinal observational study of surviving pediatric post-COVID-19 patients (n=53) and pediatric subjects without laboratory-confirmed COVID-19 included as controls (n=52) was performed.

**RESULTS:**

The median duration between COVID-19 diagnosis (n=53) and follow-up was 4.4 months (0.8-10.7). Twenty-three of 53 (43%) patients reported at least one persistent symptom at the longitudinal follow-up visit and 12/53 (23%) had long COVID-19, with at least one symptom lasting for >12 weeks. The most frequently reported symptoms at the longitudinal follow-up visit were headache (19%), severe recurrent headache (9%), tiredness (9%), dyspnea (8%), and concentration difficulty (4%). At the longitudinal follow-up visit, the frequencies of anemia (11% *versus* 0%, *p*=0.030), lymphopenia (42% *versus* 18%, *p*=0.020), C-reactive protein level of >30 mg/L (35% *versus* 0%, *p*=0.0001), and D-dimer level of >1000 ng/mL (43% *versus* 6%, *p*=0.0004) significantly reduced compared with baseline values. Chest X-ray abnormalities (11% *versus* 2%, *p*=0.178) and cardiac alterations on echocardiogram (33% *versus* 22%, *p*=0.462) were similar at both visits. Comparison of characteristic data between patients with COVID-19 at the longitudinal follow-up visit and controls showed similar age (*p*=0.962), proportion of male sex (*p*=0.907), ethnicity (*p*=0.566), family minimum monthly wage (*p*=0.664), body mass index (*p*=0.601), and pediatric pre-existing chronic conditions (*p*=1.000). The Pediatric Quality of Live Inventory 4.0 scores, median physical score (69 [0-100] *versus* 81 [34-100], *p*=0.012), and school score (60 [15-100] *versus* 70 [15-95], *p*=0.028) were significantly lower in pediatric patients with COVID-19 at the longitudinal follow-up visit than in controls.

**CONCLUSIONS:**

Pediatric patients with COVID-19 showed a longitudinal impact on HRQoL parameters, particularly in physical/school domains, reinforcing the need for a prospective multidisciplinary approach for these patients. These data highlight the importance of closer monitoring of children and adolescents by the clinical team after COVID-19.

## INTRODUCTION

The clinical and laboratory spectrum of coronavirus disease 2019 (COVID-19) in pediatric populations ranges from asymptomatic infection to acute severe conditions, such as pediatric severe acute respiratory syndrome (SARS) and multisystem inflammatory syndrome in children (MIS-C) (1-8). Despite its potential critical outcome, the rates of survival in children and adolescents with this emerging infectious disease range from 97% to 99.9% (1-10).

The COVID-19 pandemic caused by severe acute respiratory syndrome coronavirus-2 (SARS-CoV-2) infection continues to impact children and adolescents worldwide (1). The burden of this global health pandemic on pediatric populations is wide, with physical, social, emotional, and learning impacts (5,10,11).

These findings seem to be more relevant for pediatric COVID-19 survivors than for children and adolescents not infected with SARS-CoV-2. Indeed, signs and symptoms may be persistent and incapacitating after pediatric and adult COVID-19, named post-acute sequelae of COVID-19 (PASC) (12-18). Long-term PASC or long COVID-19 manifestations are defined as clinical abnormalities continuing after 12 weeks of the onset of acute COVID-19 and cannot be justified by other conditions (12,14-17,19,20).

Longitudinal studies evaluating pediatric COVID-19 survivors were conducted for less than 30 days to 9 months after the onset of acute COVID-19, and are limited to case reports or case series on cohorts from Australia, Italy, Israel, Sweden, Switzerland, United Kingdom, and United States of America (13-17,19,21,22). To the best of our knowledge, no study has evaluated persistent manifestations and health-related quality of life (HRQoL) parameters in pediatric COVID-19 survivors, particularly including predominance of previous chronic conditions in a pediatric population attending a tertiary referral hospital in Latin America.

Therefore, the objectives of the present study were to prospectively assess demographic, anthropometric, and clinical data, as well as laboratory tests, imaging abnormalities, treatments, and outcomes in symptomatic pediatric patients with laboratory-confirmed COVID-19. Demographic, anthropometric, and HRQoL data were also compared between pediatric laboratory-confirmed COVID-19 at the longitudinal follow-up visit and pediatric patients without laboratory-confirmed COVID-19 (control group).

## METHODS

This was a prospective observational study of pediatric post-COVID-19 survivors with laboratory-confirmed COVID-19. The inclusion criteria were as follows: symptomatic inpatients and outpatients, laboratory-confirmed SARS-CoV-2 infection, and age between 8 and 18 years. The exclusion criteria were subjects with asymptomatic disease and those who did not complete the Pediatric Quality of Live Inventory 4.0 (PedsQL 4.0) because of severe cognitive dysfunction and incomplete PedsQL data.

SARS-CoV-2 infection was assessed by real-time reverse transcription-polymerase chain reaction (real-time RT-PCR) or antibody testing. Real-time RT-PCR to evaluate SARS-CoV-2 RNA was performed on swab-collected nasopharyngeal and/or oropharyngeal samples from the Molecular Biology Laboratory of our tertiary hospital (23). Antibodies against S proteins from the coronavirus spike were obtained from the Laboratório de Imunologia do Instituto Central do Hospital das Clínicas da Faculdade de Medicina da Universidade de São Paulo (HCFMUSP) using two assays: enzyme-linked immunosorbent assay for anti-SARS-CoV-2 IgG antibodies and rapid immunochromatographic assay for anti-SARS-CoV-2 IgM and IgG antibodies (24,25).

Between April 11, 2020, and August 10, 2021, 151 symptomatic and asymptomatic pediatric COVID-19 survivors with laboratory-confirmed COVID-19 were invited to participate. Of these, 95 pediatric patients with COVID-19 agreed to participate in this study, and 42 were excluded (n=27, <8 years; n=11, asymptomatic pediatric COVID-19; n=2, severe cognitive dysfunction; and n=2, incomplete PedsQL data). Therefore, the final study group included 53 patients with symptomatic pediatric COVID-19. The first patient’s longitudinal visit in the present study was on October 14, 2020.

From April 4, 2021, to August 10, 2021, 61 pediatric subjects without laboratory-confirmed COVID-19 were included in the control group. The control group was recruited from the outpatient clinic of the same university hospital during routine visits. The control group were balanced in terms of age, sex, and pediatric pre-existing chronic conditions. The inclusion criteria were as follows: subjects without warning signs of pediatric COVID-19, as previously reported (4), age between 8 and 18 years, and both negative polymerase chain reaction for SARS-CoV-2 and antibody-based SARS-CoV-2 analysis at study entry. Of these subjects, 9 were excluded because of their current age being below 8 years. The final control group comprised 52 subjects.

Data were prospectively captured by the Research Electronic Data Capture database of information for all subjects at the HCFMUSP. The study was approved by the ethics committee of our university hospital (CAEE 4.889.659) and informed consent was obtained from all the participants or their parents/guardians.

### PedsQL instrument

HRQoL was analyzed using the PedsQL. This instrument was previously validated for Portuguese language and includes 23 items and 4 domains: physical functioning (8 items), emotional functioning (5 items), social functioning (5 items), and school functioning (5 items) (26). Both children and adolescents with laboratory-confirmed COVID-19 and controls completed self-report instruments for ages between 8 and 18 years. The instructions asked how much of a problem each item had been in the past 1 month. A five-point response scale was used, with 0 indicating never a problem and 4 indicating almost always a problem. Items were reverse-scored and linearly transformed to a 0-100 scale (0=100, 1=75, 2=50, 3=25, and 4=0), with higher scores indicating better HRQoL. The scale scores were computed as the sum of the items divided by the number of items answered. If >50% of the responses were missing, the scale score was not computed (26). The physical health summary score (eight items) was the same as that of the physical functioning subscale. The psychosocial health summary score (15 items) was computed as the sum of the items divided by the number of items answered in the emotional, social, and school functioning subscales.

### Demographic, anthropometric, and clinical data, and treatments and outcomes

Demographic data of the pediatric laboratory-confirmed COVID-19 patients included current age, sex, and ethnicity at diagnosis and/or at the longitudinal follow-up visit. Body mass index (BMI) was calculated as body weight divided by the square of the body height and expressed in units of kg/m^2^. The following clinical manifestations at pediatric COVID-19 diagnosis and/or at longitudinal follow-up visit were assessed by dichotomous questions (yes and no): fever, nasal discharge, sneezing, cough, sore throat, anosmia, dysgeusia, headache, myalgia, arthralgia, conjunctivitis, dyspnea, hypoxemia (oxygen saturation <92%, pulse oximetry), nausea, vomiting, diarrhea, cutaneous rash, neurological abnormalities, and pneumonia. The number of days of sign/symptom onset before COVID-19 diagnosis was evaluated at admission. Pediatric SARS was defined by the presence of flu-like syndrome with at least one of the following: dyspnea, oxygen saturation below 95% in room air, or signs of respiratory distress (4). MIS-C was diagnosed according to the Centers for Disease Control and Prevention criteria (27). According to Dong et al. (28), the severity of pediatric COVID-19 was classified as mild, moderate, severe, or critical.

Further analysis of pediatric COVID-19 survivors was performed at the longitudinal follow-up visit. Three additional dichotomous questions (yes and no) were included in the clinical evaluation: difficulty concentrating, tiredness, and poor sleep quality. Global assessment of health by pediatric patients with COVID-19 and by their primary caregiver was evaluated according to the visual analog scale (VAS) score at the longitudinal follow-up visit, which ranged from 0 (no impact) to 10 (severe impact).

Pediatric pre-existing chronic conditions were defined according to the duration of signs and symptoms for more than 3 months, and were diagnosed based on the scientific knowledge of physicians, valid techniques, or tools according to specific pediatric diagnostic criteria (29-31).

Laboratory tests for COVID-19 included assessment of hemoglobin concentration; leukocyte, neutrophil, lymphocyte, and thrombocytes; and two inflammatory biomarkers (C-reactive protein and D-dimer). These examinations were performed at the Instituto Central do HCFMUSP and Laboratory of Medical Investigation (LIM-36). Lung computed tomography and chest radiography were performed by the same pediatric radiologist at Instituto da Criança e do Adolescente do HCFMUSP, who searched for abnormalities suggestive of SARS-CoV-2-related lesions, such as ground-glass opacities, consolidation, vascular ingurgitation, septal and/or peribronchial thickening, nodules, consolidation, and hyperinflation. Echocardiographic abnormalities were evaluated by two pediatric specialists in Instituto da Criança e do Adolescente do HCFMUSP, who searched for abnormalities suggestive of SARS-CoV-2-related lesions, such as myocardial dysfunction, pericarditis, coronary artery aneurysm z-scores of ≥2.5, and valvulitis.

Treatments for acute COVID-19, including blood product transfusion (red blood cells, platelets, and plasma), oxygen therapy, concomitant antibiotics, oseltamivir, intravenous immunoglobulin, enoxaparin, aspirin, glucocorticoid, intravenous methylprednisolone pulse therapy, dialysis, current immunosuppressive therapy, and chemotherapy for patients with cancer, were also recorded. Outcomes of pediatric patients with COVID-19 who were admitted to the hospital were also evaluated, including hospitalization, duration of ward admission, duration of pediatric intensive care unit hospitalization, mechanical ventilation, duration of mechanical ventilation, vasoactive agent use, arterial hypotension, shock, disseminated intravascular coagulation, and thrombosis.

### Statistical analysis

Statistical analyses were performed using SPSS software, version 22 (IBM Corporation, Armonk, NY, USA). Non-parametric tests (Mann-Whitney test) or parametric tests (Student’s t-test) were performed for continuous variables and are presented as median (minimum and maximum values) or mean±standard deviation, respectively. Fisher’s exact test or chi-square test was performed for categorical variables. The level of significance was established at 5%.

## RESULTS

The median duration between COVID-19 diagnosis (n=53) and longitudinal follow-up visit was 4.4 months (0.8-10.7). Symptoms resolved after COVID-19 diagnosis in 30/53 (57%) patients. At least one persistent symptom was reported at the longitudinal follow-up visit in 23/53 (43%) patients, who were classified as having mild (16/23 [70%]) or moderate/severe/critical pediatric COVID-19 (7/23 [30%]). The median duration of persistent symptoms in these 23 patients after pediatric COVID-19 diagnosis was 3 months (1-10.7). Furthermore, 12/53 (23%) pediatric patients with COVID-19 reported at least one symptom lasting beyond 12 weeks and were classified as having long-term PASC or long COVID-19.

Table 1 presents the demographic, anthropometric, and clinical data, as well as pediatric pre-existing chronic conditions and comorbidities in children and adolescents with laboratory-confirmed COVID-19 at diagnosis and the longitudinal follow-up visit. The most frequently reported symptoms at longitudinal follow-up visits in the pediatric laboratory-confirmed COVID-19 patients were headache (19%), severe recurrent headache (9%), tiredness (9%), dyspnea (8%), difficulty concentrating (4%), myalgia (4%), arthralgia (4%), and poor sleep quality (4%). Two of five patients who reported severe recurrent headache at longitudinal follow-up stated that these symptoms started only after COVID-19 diagnosis and did not present during the acute phase of the disease. At the longitudinal follow-up visit, the median global assessment of health according to VAS by patients was 1.9 (0-10), and by primary caregivers was 2.2 (0-10) (Table 1).

At the longitudinal follow-up visit, cardiac, gastrointestinal, renal, and hematological manifestations resolved in 2/3 patients with MIS-C. One patient with MIS-C, without any pre-existing chronic condition, had persistent arterial hypertension after diagnosis and required antihypertensive treatment. Another patient had previous diagnosis of neurofibromatosis type 1 and required nocturnal bilevel positive airway pressure (BiPAP); she had pediatric SARS with persistent dyspnea after COVID-19 diagnosis, necessitating increase in BiPAP for continuous use over 24h.

Table 2 illustrates the laboratory tests and imaging abnormalities in pediatric laboratory-confirmed COVID-19 at diagnosis and at the longitudinal follow-up visit. The frequencies of anemia (11% *versus* 0%, *p*=0.030), lymphopenia (42% *versus* 18%, *p*=0.020), C-reactive protein level of >30 mg/L (35% *versus* 0%, *p*=0.0001), and D-dimer level of >1000 ng/mL (43% *versus* 6%, *p*=0.0004) were significantly higher between COVID-19 diagnosis and the longitudinal follow-up visit. Chest X-ray abnormalities (11% *versus* 2%, *p*=0.178) and cardiac alterations on echocardiogram (33% *versus* 22%, *p*=0.462) were similar in both groups; however, the incidence of pericarditis was significantly higher in the COVID-19 diagnosis group (25% *versus* 0%, *p*=0.013) (Table 2).

Table 3 shows the demographic and anthropometric data and PedsQL 4.0 scores of pediatric laboratory-confirmed COVID-19 at longitudinal follow-up visit and in controls (pediatric patients without laboratory-confirmed COVID-19). The median current age (14.65 [8-18] *versus* 14.84 [8-18] years, *p*=0.962), family minimum monthly wages (2 [0.2-10] *versus* 2 [0-8], *p*=0.664), and BMI (20.2 [14.5-39.5] *versus* 19.6 [13.8-34.9] kg/m^2^, *p*=0.601) were similar in the patient and control groups. Likewise, the frequencies of male sex (42% *versus* 40%, *p*=0.907), pediatric pre-existing chronic conditions (89% *versus* 90%, *p*=1.000), ethnicity (*p*=0.566), and maternal level of schooling (*p*=0.855) were similar in both groups. Regarding PedsQL 4.0 scores, the median physical score (69 [0-100] *versus* 81 [34-100], *p*=0.012) and school score (60 [15-100] *versus* 70 [15-95], *p*=0.028) were significantly lower in the pediatric laboratory-confirmed COVID-19 patients than in the control group. No significant differences were evidenced in any pediatric chronic condition between the groups (*p*>0.05). The crucial pediatric chronic diseases were immunosuppressive conditions, with similar frequencies in both groups (83% *versus* 77%, *p*=0.473) (Table 3).

## DISCUSSION

We demonstrated that approximately 40% of patients in the pediatric laboratory-confirmed COVID-19 patients reported at least one persistent symptom at the longitudinal follow-up visit, and one-fourth had long COVID-19. Further, pediatric patients with COVID-19 showed a longitudinal impact on HRQoL parameters, particularly in the physical and school domains.

The main strength of the present prospective study is the inclusion of children and adolescents with laboratory-confirmed SARS-CoV-2 infection, involving approximately 90% of subjects with multicomplex pre-existing chronic conditions followed up in a tertiary university hospital (29,30). Another strength was the inclusion of a control group without laboratory-confirmed SARS-CoV-2 infection at study entry, balanced by age, sex, ethnicity, socioeconomic status, BMI, and pediatric chronic illnesses. These findings are relevant as each of these variables may influence HRQoL parameters (32-35). Another advantage of the present study is the multidisciplinary and multiprofessional team supervision for studies of pediatric COVID-19 survivors, supporting several prospective projects that were simultaneously proposed and ethically approved at HCFMUSP.

Importantly, a new outpatient clinic has been organized at Instituto da Criança e do Adolescente do HCFMUSP, with visits for each pediatric COVID-19 survivor every 6 months. Longitudinal projects for this population include prospective evaluations of sociodemographic data, linear growth, pubertal development, clinical, nutritional, laboratorial, and imaging examinations; mental health status; environmental exposure; errors innate to immunity; autoimmunity; metabolomics; gut microbiota; genetics; bone metabolism; and physical activity studies. Further prospective analyses of cardiovascular imaging and physical activity program implementation are crucial for patients with MIS-C and pediatric SARS.

We confirmed that at least 40% of pediatric patients with COVID-19 reported at least one symptom at the longitudinal follow-up visit, whereas previous studies have reported rates of 8%-66% (13-16,19,21). In addition, the incidence of long-term PASC or long COVID-19 has been reported to range from 0% to 27%, similar to the current observations (14-17,19,20). A Swiss study showed that the most frequently reported symptoms associated with long-term PASC in children and adolescents were tiredness (3%), difficulty concentrating (2%), and poor sleep quality (2%) (16). Long-term complications can also occur in pediatric patients with mild COVID-19, as observed in the present study (15).

Furthermore, long COVID-19 may occur in critical cases of MIS-C and pediatric SARS. A United Kingdom study demonstrated that cardiac, gastrointestinal, renal, and hematological manifestations in patients with MIS-C resolved at 6 months, and emotional difficulties were common (19). These findings were not observed in our patients with MIS-C, and these patients had no difficulty concentrating on reports at the longitudinal follow-up visit. However, one patient presented with sustained arterial hypertension after MIS-C diagnosis, as assessed 4 months after acute infection-related hospitalization, which was sustained for almost 1 year after this event.

We herein extend previous reports of laboratory-confirmed pediatric COVID-19 (19) with predominance of pre-existing chronic conditions, demonstrating an impact on HRQoL parameters, specifically in the physical and school domains. Physical impact may be associated with musculoskeletal involvement (fatigue and muscle weakness) after pediatric COVID-19, a common finding after acute infection, whereas school impact may be related to cognitive dysfunction after SARS-CoV-2 infection; these are now regularly studied in our pediatric COVID-19 patient cohort. Thus, we propose that physical activity programs involving physiotherapy and mental health support are crucial in mitigating the prolonged effects of COVID-19 in the pediatric population (6,36,37).

Most pediatric COVID-19 survivors had good physical and functional recovery during the first 4 months of follow-up, reinforcing the notion that the majority of children and adolescents respond quickly after infection. However, patients with persistent symptoms may experience difficulties that affect their quality of life. In contrast, a Chinese study on adult COVID-19 survivors showed that at least one symptom was observed in 68% of patients at 6 months and in 49% of patients after 12 months (19).

This study has some limitations. It was carried out at a single center in a large metropolitan tertiary pediatric hospital, and mainly included children and adolescents with a more severe spectrum of SARS-CoV-2 infection. Moreover, we only assessed the first visit after a median of 4 months of acute COVID-19; therefore, long-term health consequences studies of laboratory-confirmed COVID-19 in children and adolescents are required.

In conclusion, pediatric patients with COVID-19 demonstrate a longitudinal impact on HRQoL parameters, specifically in the physical and school domains. These data highlight the importance of close monitoring of children and adolescents by the clinical team after COVID-19.

### HC-FMUSP Pediatric Post-COVID-19 Study Group

Adriana M. E. Sallum, Alexandra V. M. Brentani, Álvaro José S. Neto, Amanda Ihara, Andrea R. Santos, Ana Pinheiro M. Canton, Andreia Watanabe, Angélica C. dos Santos, Antonio C. Pastorino, Bernadette D. G. M. Franco, Bruna Caruzo, Carina Ceneviva, Carolina C. M. F. Martins, Danilo Prado, Deipara M. Abellan, Fabiana B. Benatti, Fabiana Smaria, Fernanda T. Gonçalves, Fernando D. Penteado, Gabriela S. F. de Castro, Guilherme S. Gonçalves, Hamilton Roschel, Ilana R. Disi, Isabela G. Marques, Inar A. Castro, Izabel M. Buscatti, Jaline Z. Faiad, Jarlei Fiamoncini, Joaquim C. Rodrigues, Jorge D. A. Carneiro, Jose A. Paz, Juliana C. Ferreira, Juliana C. O. Ferreira, Katia R. Silva, Karina L. M. Bastos, Katia Kozu, Lilian M. Cristofani, Lucas V. B. Souza, Lucia M. A. Campos, Luiz Vicente R. F. Silva Filho, Marcelo T. Sapienza, Marcos S. Lima, Marlene P. Garanito, Márcia F. A. Santos. Mayra B. Dorna, Nadia E. Aikawa, Nadia Litvinov, Neusa K. Sakita, Paula V. V. Gaiolla, Paula Pasqualucci, Ricardo K. Toma, Simone Correa-Silva, Sofia M. Sieczkowska, Marta Imamura, Silvana Forsait, Vera A. Santos, Yingying Zheng. All investigators are from Hospital das Clinicas HCFMUSP, Faculdade de Medicina, Universidade de Sao Paulo, SP, BR.

## AUTHOR CONTRIBUTIONS

All named authors have approved the final draft of the manuscript and its submission to the journal, and are willing to take responsibility for it in its entirety. All authors contributed substantially to the conception and design of the study, and in the analysis and interpretation of data. All authors revised the manuscript critically and approved its final version.

## Figures and Tables

**Table 1:** Demographic, anthropometric, and clinical data, and treatments and outcomes in pediatric laboratory-confirmed coronavirus disease (COVID-19) at diagnosis and longitudinal follow-up visit.

Variables	Diagnosis (n=53)	Follow-up (n=53)
Demographic and anthropometric data		
Current age (years)	14.65 (8-18)	-
Time between diagnosis and study entry (months)	-	4.4 (0.8-10.7)
Male sex	22 (42)	-
Caucasians	30 (57)	-
Clinical data		
Duration of signs/symptoms before diagnosis (days)	3 (1-30)	-
Fever	33 (62)	0 (0)
Duration of fever (days)	3 (1-22)	-
Nasal discharge	24 (45)	0 (0)
Sneezing	7 (13)	0 (0)
Cough	27 (51)	0 (0)
Sore throat	16 (30)	0 (0)
Anosmia	13 (25)	0 (0)
Dysgeusia	11 (21)	0 (0)
Headache	30 (57)	10 (19)
Severe recurrent headaches	0 (0)	5 (9)
Myalgia	21 (40)	2 (4)
Arthralgia	8 (15)	2 (4)
Conjunctivitis	5 (9)	0 (0)
Dyspnea	14 (26)	4 (8)
Hypoxemia	5 (9)	1 (2)
Nausea	17 (32)	0 (0)
Vomiting	15 (28)	0 (0)
Diarrhea	15 (28)	0 (0)
Abdominal pain	13 (25)	1 (2)
Cutaneous rash	6 (11)	1 (2)
Neurological abnormalities	1 (2)	1 (2)
Pneumonia	4 (8)	0 (0)
Severe acute respiratory syndrome	3 (6)	0 (0)
Multisystem inflammatory syndrome in children (MIS-C)	3 (6)	-
Difficulty concentrating	-	2 (4)
Tiredness	-	5 (9)
Poor sleep quality	-	2 (4)
Pediatric pre-existing chronic conditions	47 (89)	-
Diabetes mellitus	1 (2)	-
Arterial hypertension	2 (4)	-
Immunosuppressive conditions	44 (83)	-
Inborn errors of immunity	0 (0)	-
Transplantation	5 (9)	-
Cancer	7 (13)	-
Chronic kidney disease	3 (6)	-
Autoimmune conditions	14 (26)	-
Others pre-existing chronic conditions	24 (45)	-
Pediatric COVID-19 classification		
Mild	41 (77)	-
Moderate	6 (11)	-
Severe	3 (6)	-
Critical	3 (6)	-
Global assessment of health by VAS score (0-10)		
Patient	-	1.9 (0-10)
Primary caregiver	-	2.2 (0-10)
Treatments		
Blood products transfusion	2 (4)	0 (0)
Oxygen therapy	5 (9)	1 (2)
Intravenous immunoglobulin	3 (6)	0 (0)
Enoxaparin	4 (8)	1 (2)
Glucocorticoid	5 (9)	0 (0)
Dialysis for acute renal injury or shock	0 (0)	0 (0)
Aspirin	3 (6)	2 (4)
Currently immunosuppressive	24 (45)	24 (45)
Outcomes		
Hospitalization	18 (38)	-
Duration of ward admission, days (n=18)	5 (1-22)	-
Pediatric intensive care unit hospitalization	4 (22)	-
Duration of PICU hospitalization, days (n=4)	6.5 (6-11)	-
Mechanical ventilation	2 (4)	-
Vasoactive agents use	3 (4)	-
Disseminated intravascular coagulation	1 (2)	-
COVID-19-associated thrombosis	1 (2)	1 (2)

Results are presented as n (%) or median (minimum-maximum values). VAS: Visual analog scale, PICU: Pediatric intensive care unit.

**Table 2:** Laboratory tests and imaging abnormalities in pediatric laboratory-confirmed coronavirus disease (COVID-19) cases at diagnosis and longitudinal follow-up visit.

Variables	Diagnosis (n=53)	Follow-up (n=53)	*p*
Hematological parameters			
Anemia, hemoglobin <10 g/dL	4/36 (11)	0/51 (0)	0.030
Leucopenia <4,000/mm^3^	7/36 (19)	7/51 (14)	0.560
Neutropenia <1,000/mm^3^	1/36 (3)	2/51 (4)	1.000
Lymphopenia <1,500/mm^3^	15/36 (42)	9/51 (18)	0.020
Thrombocytopenia <100,000/mm^3^	5/36 (14)	3/51 (6)	0.270
Inflammatory markers			
C-reactive protein >30 mg/L	12/34 (35)	0/51 (0)	0.0001
D-dimer >1000 ng/mL	9/21(43)	3/51 (6)	0.0004
Lung computed tomography abnormalities	4/8 (50)	0/0 (0)	-
Ground-glass opacities	3/8 (37)	0/0 (0)	1.000
Vascular ingurgitation	1/8 (12)	0/0 (0)	1.000
Peribronchial thickening	3/8 (37)	0/0 (0)	1.000
Septal thickening	1/8 (12)	0/0 (0)	1.000
Small nodules	1/8 (12)	0/0 (0)	1.000
Chest X-ray abnormalities	2/18 (11)	1/48 (2)	0.178
Consolidation	2/18 (11)	0/48 (0)	0.071
Hyperinflation	0/18 (0)	1/48 (2)	1.000
Cardiac alterations by echocardiogram	4/12 (33)	8/36 (22)	0.462
Myocardial dysfunction	2/12 (17)	8/36 (22)	1.000
Pericarditis	3/12 (25)	0/36 (0)	0.013
Coronary artery aneurism z-scores ≥2.5	1/12 (8)	0/36 (0)	0.250
Valvulitis	0/12 (0)	1/36 (3)	1.000

Results are presented as n (%).

**Table 3:** Demographic, anthropometric, and Pediatric Quality of Life Inventory 4.0 (PedsQL 4.0) scores in subjects with pediatric laboratory-confirmed coronavirus disease (COVID-19) at longitudinal follow-up visit and in controls (subjects without laboratory-confirmed COVID-19).

Variables	Pediatric laboratory-confirmed COVID-19 patients (n=53)	Control group (n=52)	*p*
Demographic			
Current age (years)	14.65 (8-18)	14.84 (8-18)	0.962
Male sex	22 (42)	21 (40)	0.907
Ethnicity			
Caucasian	30 (57)	25 (48)	0.566
African-Latin American	21 (49)	25 (48)	
Asian	1 (2)	0 (0)	
Others/unknown	1 (2)	2 (4)	
Social assistance program	14/26 (54)	16/37 (43)	0.407
Family minimum wages/month	2 (0.2-10)	2 (0-8)	0.664
Number of household’s members in the residence	4 (2-6)	4(2-8)	0.690
Mother current age (years)	43.3 (31.6-60.9)	45 (27.8-55.4)	0.471
Mother's level of schooling (n=49)			
Elementary school	7 (14)	7 (14)	0.855
Middle school	8 (16)	5 (10)	
High school	19 (39)	20 (41)	
University	15 (31)	17 (35)	
Anthropometric data			
Body mass index (kg/m^2^)	20.2 (14.5-39.5)	19.6 (13.8-34.9)	0.601
Pediatric pre-existing chronic conditions	47 (89)	47 (90)	1.000
Diabetes mellitus	1 (2)	1 (2)	1.000
Arterial hypertension	2 (4)	1 (2)	1.000
Immunosuppressive conditions	44 (83)	40 (77)	0.473
Inborn errors of immunity	0 (0)	0 (0)	1.000
Transplantation	5 (9)	9 (17)	0.264
Cancer	7 (13)	3 (6)	0.319
Chronic kidney disease	3 (6)	2 (4)	1.000
Autoimmune conditions	14 (26)	9 (17)	0.346
Others pre-existing chronic conditions	24 (45)	30 (58)	0.243
PedsQL 4.0 scores			
Total score (0-100)	69.1 (10-99)	52 (31-98)	0.053
Physical (0-100)	69 (0-100)	81 (34-100)	0.012
Psychosocial (0-100)	67 (20-100)	72 (25-98)	0.362
Emotional (0-100)	60 (0-100)	55 (10-100)	0.741
Social (0-100)	85 (20-100)	80 (5-100)	0.966
School (0-100)	60 (15-100)	70 (15-95)	0.028

Results are presented as n (%) or median (minimum-maximum values) and mean±standard deviation.
